# Bridging the Heterogeneity of Myasthenia Gravis Scores as a Foundational Step Towards the Construction of a Digital Twin

**DOI:** 10.3390/biomedicines13122920

**Published:** 2025-11-28

**Authors:** Marc Garbey, Quentin Lesport, Henry J. Kaminski

**Affiliations:** 1 Care Constitution Corp., Houston, TX 77057, USA; lesport@email.gwu.edu; 2 LaSIE UMR-CNRS 7356, University of La Rochelle, 17042 La Rochelle, France; 3 Department of Neurology & Rehabilitation Medicine, George Washington University, Washington, DC 20037, USA

**Keywords:** precision medicine, digital twin, myasthenia gravis, ptosis, diplopia, muscular weakness, neurological disease, clinical trial, virtual population

## Abstract

**Background/Objectives**: Myasthenia gravis (MG) is a rare autoimmune neuromuscular disease. Clinical trials with rigorously collected data provide valuable opportunities for mathematical modeling of patient outcomes over time. However, for rare diseases such as MG, combining data across multiple trials presents challenges due to heterogeneity in outcome measures. This study aims to address these challenges by investigating relationships among commonly used MG outcome measures to support the development of a standardized “Myasthenia Gravis Portrait.” **Methods**: We integrated three primary data types from multiple clinical studies: (i) laboratory and medication data, (ii) Electronic Health Record (EHR) data (e.g., age, sex, years since diagnosis, BMI), and (iii) disease severity scores. We examined the relationships among several MG-specific scoring systems, including Activities of Daily Living (MG-ADL), Quantitative Myasthenia Gravis (QMG), MG Composite (MG-CE), and MG Quality of Life-15 (MGQOL-15), to evaluate consistency and comparability across studies. **Results**: Preliminary analyses revealed variable correlations among the different scoring systems, indicating that, while some measures capture overlapping aspects of disease progression, others reflect distinct patient- or clinician-centered perspectives. These findings highlight the need for a harmonized framework that captures both functional and clinical dimensions of MG severity. **Conclusions**: The proposed “Myasthenia Gravis Portrait” provides a standardized approach for representing patient outcomes across diverse clinical datasets. This framework will facilitate the creation of virtual populations of digital twins, enabling a machine-learning-based modeling of MG progression and prediction of individualized disease trajectories.

## 1. Introduction

Myasthenia gravis (MG) is a rare, autoimmune, neuromuscular disorder caused primarily by antibodies directed towards the acetylcholine receptor (AChR) on the post-synaptic surface of the neuromuscular junction, leading to fluctuating weakness manifest as disabling visual symptoms to life-threatening respiratory failure [1]. There is a significant unmet need, with poor adverse effect profiles of therapies, wide variation in responses, and a third of patients being treatment-resistant despite recent FDA-approved medications having a poorer-than-expected benefit in common clinical practice.

Validated clinical outcome measures used to assess MG patients have been established, with some being based on symptoms reported by the patient, others by clinical examination, and some are hybrid. We utilized the accepted clinical outcome measures for this study:

- Quantitative Myasthenia Gravis (QMG) score: The QMG is based on physical examination of sentinel muscle groups compromised by MG [2,3].
- Myasthenia Gravis Activities of Daily Living (MG-ADL) scale: This patient-reported outcome measure assesses MG symptoms and functional activities related to daily living. The score is the primary outcome measure used by the FDA for drug approval [4,5].
- Myasthenia Gravis Quality of Life-15 item scale (MG-QOL15): This patient self-reported scale assesses the patient’s daily functioning, well-being, and overall quality of life, including fatigue in patients, which is a common complaint [6,7].
- The new MG-MGCE score was developed to adapt the neuromuscular examination for performance during telemedicine evaluations [8].

A common feature of all these scores is the heuristic nature of the data acquisition and potential bias due to the method of reporting and/or observation. The QMG and MG-ADL scores have essential differences: the QMG provides a quantitative measure of muscle strength at a single point in time performed by a trained investigator or research coordinator. The MG-ADL provides a measure of the patient’s perception in performing activities in the previous two weeks of evaluation [9]. The Myasthenia Gravis Composite (MGC) score is based on physician examination and patient reported impairment in performance of specific activities [10,11]. The MGC score is a simple combination of both MG-ADL and QMG scores in an attempt to provide a comprehensive assessment of MG severity, combining physician-assessed examinations (evaluates ocular, neck, and proximal limb muscles) and patient-reported outcomes (assesses speech, chewing, swallowing, and respiratory function). Our objective is to move beyond this basic score combination to generate a more nuanced and insightful clinical score portrait from multiple disparate clinical studies as a foundational step toward a future digital twin of the patient.

In this paper, we use data sets from three clinical studies: the ADAPT-TeleMG data set [8,12,13], MGTX trial [14,15], and BeatMG trial [16] to build a clinical portrait that can be used as a basis in a common construction of a virtual population of digital twins (VPTD) [17].

Each of these trials had different clinical endpoints and goals. Therefore, it is not surprising that their population distributions obey different criteria. Clinical studies do not use the same standard score. Since MG is a rare disease and clinical trial recruitments range from fewer than 50 to 200, it will be essential to combine multiple studies to design a digital twin of the MG patient that would have the statistical power to support disease prediction. Consequently, our goal is to consolidate whatever score is available from a study into a single clinical portrait of the patient.

First, we will look within each score at its components and their relationship to each other. Janssen et al. [4] provide a thorough statistical analysis of this problem for the MG-ADL score. Second, we will look at the potential correlation across outcome measures for corresponding muscle group evaluations. Our focus is to build stochastic mapping components from one score to another, to build a “portrait” of the subject and fill potential gaps across clinical trials. A patient’s MG score portrait should be a comprehensive assessment of the disease’s severity and impact on their daily life. We construct a consolidated score from disparate incomplete ones using simple techniques including clustering and descriptive statistical methods. Together, these components paint a clinical picture of a patient’s disease and would assist healthcare providers in tailoring a treatment plan, assess treatment effectiveness, and provide prognostic information. In our previous study [18], we used the MGTX trial to develop a modeling framework aimed at enhancing the efficiency and predictive power for clinical trials, using MG as a representative example of a rare disease. This study intends to allow the generalization of this work to a larger number of clinical trials by providing a much broader base of patient data.

## 2. Materials and Methods

Datasets:

- ADAPT-teleMG (NCT05917184): We utilized a bank of 54 subjects with MG, each having two telemedicine examinations by neuromuscular experts with recording of the MG-CE score, the MG-ADL, MG-Composite Virtual, MG-CE, and Neuro_QOL15 score [7]. The total number of records was 104 in each category. A critical review of the human factors influencing the examination scores is provided in [13].
- BeatMG (B-cell depletion in MG) [16]: The phase 2 trial evaluated the use of rituximab, a medication that depletes B cells in patients with MG who were not adequately controlled with standard treatments. Fifty-two subjects were followed for one year. The primary clinical outcome measure was based on prednisone dose and MGC score. The total number of MG-ADL and QMG scores accumulated over all patients over time was 728.
- MGTX (thymectomy trial in non-thymomatous MG patients receiving prednisone therapy) [14,15]: This 3-year trial investigated the effectiveness of thymectomy plus prednisone versus prednisone alone in patients with generalized MG. Subjects were 18 to 65 years and were AChR-antibody positive. Subjects performing poorly had escalation of prednisone, and for severe worsening, were treated with rescue therapies of IVIg or plasma exchange. In total, 123 subjects were followed for 36 months. The primary outcome was the integration of the prednisone dose over the time of the study. The MG-ADL and QMG scores were performed at month M0, M3, M4, M6, M9, M12, M15, …, M36. It is appreciated that evaluations cannot be performed at exactly these times. The total number of MG-ADL and QMG scores accumulated over all patients over time was 2061, with some missing entries for each of the outcome measures.

Concept:

Our construction of a patient portrait is based on establishing stochastic maps between individual test score values of the various outcome measures which assess the same group of muscles. This construction should gather the accumulated knowledge of all clinical trial data sets. This mapping should be performed in the context of possible existing correlations between individual test scores within an assessment.

To be more precise, a stochastic map P from the discrete set of scores 𝒳={1..4} to the discrete set of numbers 𝒴={1..4} is a function that assigns a conditional probability to every pair of states $\left(x,y\right),$ where x∈𝒳 and y∈𝒴. 

This probability, denoted as $P\left(y|x\right)\text{ or }P_{xy}$, represents the likelihood of ending up in state y given that the current state is x.

This concept can be readily generalized to multiple dimension sets.

For example, the construction of the two stochastic maps that project the MG-ADL double vision/eyelid drop score to the QMG ptosis/diplopia score and vice versa is based on all the clinical data sets available, which is about 2800 entries combining MGTX and BeatMG. It can be generated either by enumeration counts or machine learning.

The mathematical formulation of the clinical score portrait of a patient gathering QMG and MG-ADL information will come as a combination of discrete values from all available scores and probability distribution of score values when a score is missing. Let us assume, for example, that a patient received a standard QMG score but the corresponding MG-ADL score for the visit is missing. Using a stochastic map from QMG to ADL, we can propose to provide the MG-ADL missing record with the probability distribution corresponding to the MG-ADL score. Table 1 below gives an illustration of what the result might be for an individual score, ptosis with provided QMG score but missing MG-ADL score:

The more predictive the stochastic map is, the better the estimate of the missing score. As we build the stochastic map from all available data, we continue to improve upon the estimate as we integrate more and more clinical study data.

In situations where two test individual scores denoted A and B have demonstrated some level of correlation, we may use stochastic maps combining both individual test scores to improve the estimate.

A “two dimensional” score estimate would take the format of the following matrix, illustrated in Table 2.

The same concept applies to more than two individual test scores depending on the level of correlation of inter-individual test scores.

If the score has been given twice or more by different clinicians, based on evaluation of video records of examinations of the same patient as was the case in the ADAPT clinical study, we may have different scores from clinicians. We replace the multiple entries of the score by a probability estimate assuming either the same weight to each clinician or possibly increase the trust on one of the evaluators if there were objective criteria to do so [8,12,13].

Assuming that all the individual components of the QoL test score are strongly correlated as opposed to the MG-ADL and QMG scores, as we will demonstrate later, the construction of the stochastic map between the QoL score and MG-ADL or QMG is more complicated.

It is no surprise that the answer of a patient to a generic question such as “I am frustrated by my MG” could be related to “I have trouble eating because of MG”. This is true also for “I have trouble with my eyes because of my MG” (e.g., double vision) and “I have limited my social activity because of my MG”, to take a few examples.

We use clustering to transform individual test scores into few clusters that illustrate the patient fatigue pattern from QoL and map this pattern directly to the total score for MG-ADL and QMG or possibly its components.

Overall, we can complete the individual score of any patient enrolled in one of the clinical trials with a uniform structure for the score portrait that combines elements of the MG-ADL, QMG, and QoL scores, for example, which can serve as a basis to predict patient trajectories as reported in our preprint [18]. We construct in that preprint the concept of a predictability index that refers to the percentage of patient trajectories that obey the principle that a patient with similar “state”—including score—should evolve the same way for a short period of time at least, i.e., as described by a continuous operator of module of continuity being one. Let us assume that the MG-ADL, QMG, and QoL scores have been normalized to stay in interval [0,1]. We can refine our strategy in assembling the score portrait by using our predictability index associated with the data set to obtain the best weighted possible combination of scores in the following format:

α MG-ADL + β QMG + γ QoL, with α + β + γ = 1,

or at the level of individual test scores within MG-ADL or QMG as shown in [18].

Since the patient portrait is a probability distribution, we can readily generate a virtual population [19,20] using a Montecarlo simulation and minimum assumption. This is indeed a resampling method that does not add new diverse patient profile and works without any modeling assumption on the patient response to treatment. We will apply our patient trajectory scheme to this virtual population to refine our previous results on treatment success, azathioprine doses in particular, revealing potential new associations with age and rescue therapy [21].

To summarize, our construction follows the algorithm described next, along with its rationale.

Algorithm:

- Step 1: Each score corresponding to muscle weakness symptoms is assembled from a battery of individual test scores, generally focusing on a specific group of muscles:
  ○ Compute the correlation matrix between individual test scores of the same score for each data set and check if correlation between individual scores is weak. The concept of weak correlation is indeed somewhat fuzzy and will be defined in the Results section. If correlation between individual test scores is not weak, regroup them as a vector.
  ○ Compute the distribution of individual test scores for each score and data set.
- Step 2: Review the fatigue test score with a similar method:
  ○ Show that the correlation between individual scores is strong.
  ○ Extract a set of independent patterns with weak correlation using a clustering algorithm.
  ○ We note that of all three data sets we used, only the ADAPT data set has a QoL score, more precisely the Neuro-Qol 15 version.
- Step 3: Establish potential correlation between score tests across scores (ref step 1). Putting aside the fatigue test, revisit the graph of inter-score correlation (see Table 3 and Table 4). Leg strength/double vision/eyelid drop is the smallest common denominator between all first three scores. All other test scores might be re-grouped by domains: talking, chewing, swallowing, count to 50 (bulbar domain); breathing, single breath count (respiratory domain); impairment of ability to brush teeth or comb hair, impairment of ability to rise from a chair (limb weakness domain); and double vision, eyelid droop, eye closure (ocular domain).
- Step 4: Extend that correlation matrix to the QoL patterns (ref step 2) of QoL Scores: frustration/ocular impairment/social activity/hobbies activity/family duties/making plan/work ability/speaking/loss of autonomy/depression/walking/moving to place/overwhelmed/personal grooming.
- Step 5: Generate a virtual population with a Monte Carlo simulation starting from the stochastic portrait of each patient data set entered in the database.

Once this construction is achieved, we can study patient score trajectories during the clinical trial and use the methodology of [16] to start to transform that data set into a predictive model of the clinical trial.

## 3. Results

**Figure 1:**
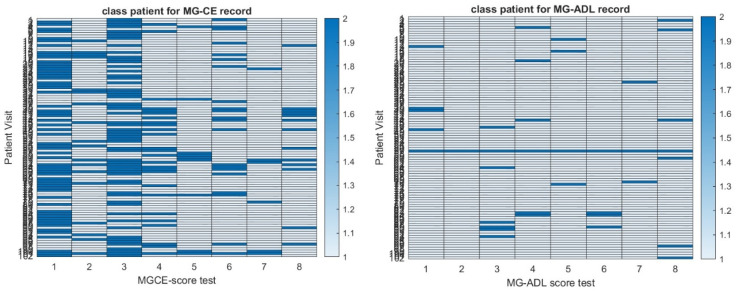
Heat map of the reproducibility of MG-CE score (**left panel**) and MG-ADL score (**right panel**) in the ADAPT-MG study as defined in [10].

We appreciate that the self-reported scores like the MG-ADL or QoL have a level of uncertainty [22] due to the subjectivity of the subject’s perception, mood, and emotional state. Further, symptoms fluctuate significantly, and the patient may develop coping strategies which lead to underestimating the severity of symptoms. Similarly, clinical examinations with QMG and MG-CE scores are not perfect and may have poor reproducibility in some categories—see Figure 1 and [13]. Score values in clinical examination scoring might become more robust with digital health measures than the common clinical practice [8,12]. For these reasons, we expect a significant level of noise and bias in our data set that could lower the robustness of the analysis.

**Figure 2:**
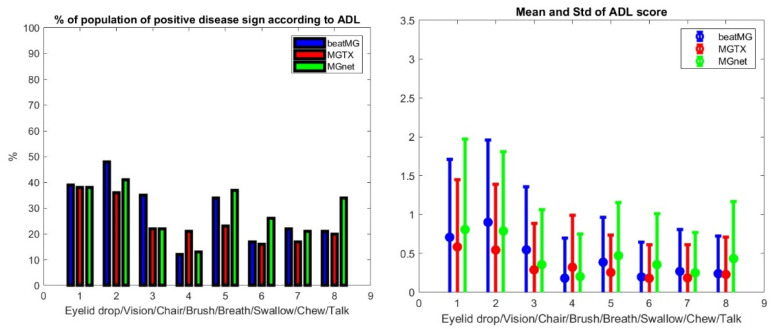
Population characteristics of the BeatMG, MGTX, and ADAPT-MG clinical studies. The **left panel** depicts the percentage of patients who had a non-normal result on MG-ADL subgroup scores in the order eyelid drop, double vision, sit to stand, brush teeth, breathing, swallowing, chewing, and talking. The **right panel** provides the corresponding average score and standard deviation of the population.

We assessed each clinical data set for the distribution of patient scores and the percentage of the population with positive symptoms using the MG-ADL for each individual score in the evaluation—see Figure 2.

We generate ρ, a generic correlation coefficient, and its *p*-values, denoted *p*. All three distributions are distinct but similar. The average scores between the three data sets have the best correlation with ρ > 0.85, *p* ≤ 0.007. It should be appreciated that, while the number of patients is roughly of the same order for each clinical trial data set, the number of registered scores we can consider as independent for our purposed evaluation is not. In fact, scores are determined at different time points of the clinical study and can be considered as independent variables, since we are only interested in correlation across scores.

Step 1: Let us first look systematically at the independence of each individual test score in MG-ADL, QMG, MG-CE, and MG-QoL15. For MG-ADL and both the MGTX and BeatMG data sets, we observe a statistically significant correlation (ρ > 0.35, *p* < 10^−10^) within the group muscle 1, i.e., eyelid drop and diplopia, as well as within the group muscle 6, i.e., chewing, swallowing, and talking along with teeth brushing. The result is less clear in the ADAPT data set but for group muscle 1. However, we appreciate that the ADAPT data set (104 records) is small compared to the two others that have multiple time points with scores:

- For QMG in both the MGTX and BeatMG data set, we observed a strong correlation (ρ > 0.83, *p* < 10^−10^) for the left arm score versus the right arm score, as well as left leg score with the right leg score. The left and right grip are correlated as well but with a lower correlation coefficient, i.e., ρ > 0.5, *p* < 10^−10^. We observe a statistically significant correlation (ρ > 0.35, *p* < 10^−10^) within group muscle 1 for both MGTX and BeatMG data sets and group muscle 6, only for the MGTX data set.
- Overall, the MGTX data set is the largest (2061 records) and gives higher correlation values than the BeatMG data set (728 records) for those individual test scores that are correlated.
- We have the MG-Qol-15 score for the ADAPT-MG data set only. As shown in Table 3, all individual test scores are statistically significantly correlated.

We regroup individual test scores that are strongly correlated, like left leg and right leg or left arm and right arm assessment in QMG, into a combined score.

It is not feasible for the fatigue test score to perform without losing critical information on the patient state and, therefore, we used an alternative approach.

**Figure 3:**
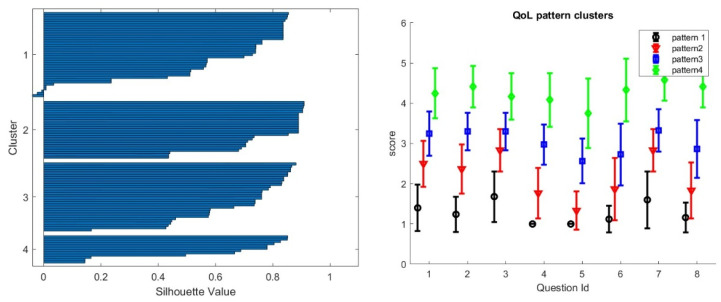
We obtain four well-separated patterns of QoL for the ADAPT-teleMG patients using a clustering algorithm. On the **left panel**, the signature silhouette shows the separation of clusters. On the **right panel,** we show the four clusters with a monotinc progression of all invidual test scores from low to high scores. The fact that the progression is monotonic across all questions is due to the strong correlation between individual test scores, as shown in Table 3.

Step 2: The Quality of Life-15 item scale—MG-QOL15—should be treated as a completely independent entity. Figure 3 shows the different pattern of fatigue obtained with four clusters that are clearly separated according to the signature function. We will present next an additional criterion to choose the optimum number of clusters besides cluster separation that is related to the construction of stochastic mapping with other clinical scores.

**Figure 4:**
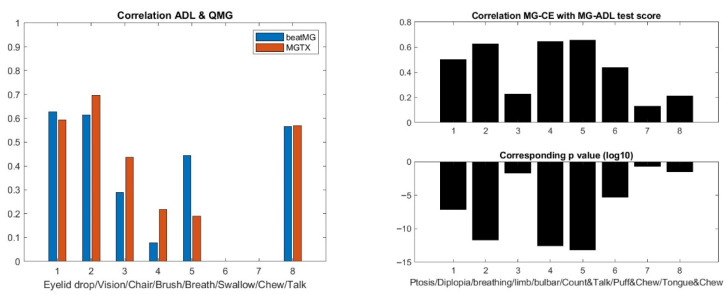
Correlation of the corresponding MG-ADL and QMG scores in the MGTX and BeatMG data sets on the **left panel** (*p*-values are far less than 10^−20^), and correlation of the corresponding MG-CE and MG-ADL scores in the ADAPT-MG study on the **right panel**.

**Figure 5:**
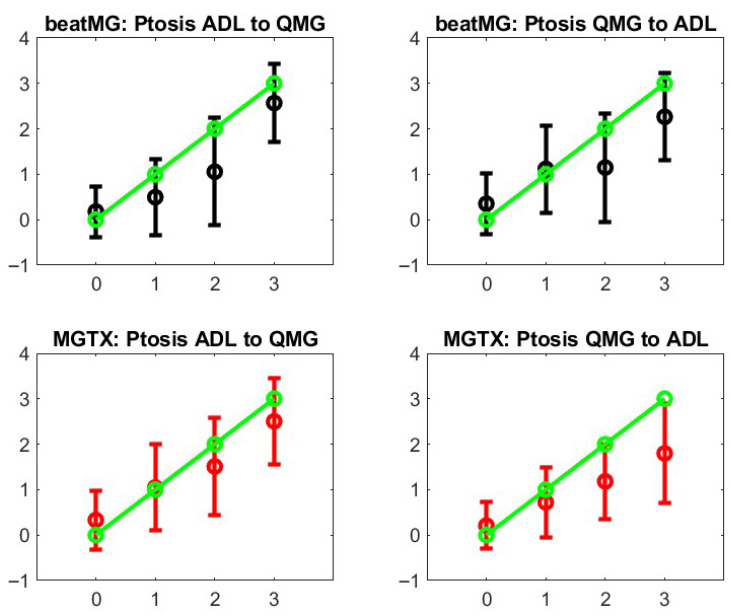
Stochastic operators from MG-ADL to QMG and their inverse. The green line represents what a perfect bijection would be. The **left panels** are for the direct map MGTX for the BeatMG and QMG data sets. The **right panels** are the corresponding inverse map.

**Figure 6:**
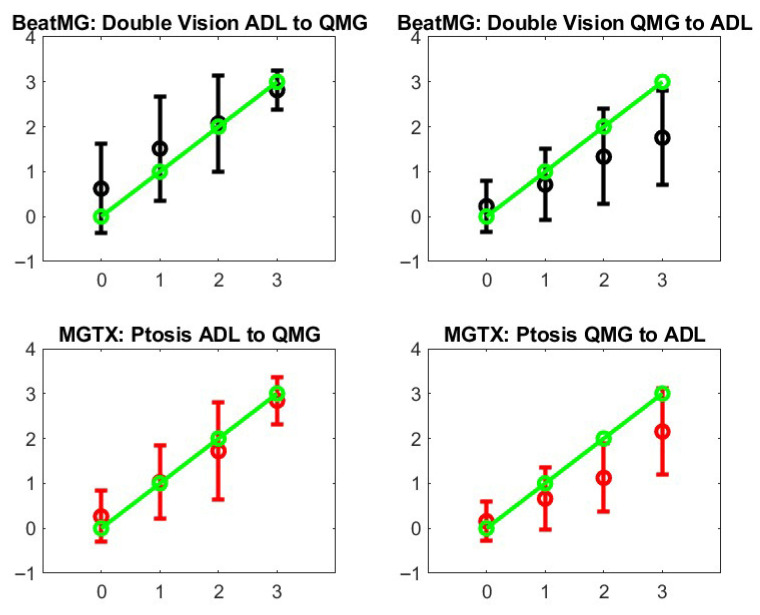
Stochastic operators from MG-ADL to QMG and their inverse for the double vision/diplopia test score. The green line represents what a perfect bijection should be. The **left panels** are for the direct map MGTX for the BeatMG and QMG data sets. The **right panels** are for the corresponding inverse map.

Step 3: We review the stochastic maps that we have built across outcome measures:

- One can map MG-ADL and QMG component by component when there is clear association, i.e., same muscle group, like ptosis, diplopia, leg strength, etc., that are scored for both MG-ADL and QMG. A similar analysis can be performed for MG-ADL and MG-CE with the ADAPT-MG data set. However, such maps would only be appropriate if the correlation were meaningful—see Figure 4. One can compare the individual test scores of MG-ADL and QMG only for ptosis, eyelid droop, and talking. On the other hand, one can compare the MG-ADL and MG-CE score only for ptosis, eyelid drop, limb, and bulbar strength. We discard the cheek puff test from the MG-CE data set because of its poor accuracy, as appreciated previously [12,13].
- Figure 5 shows the results for ptosis assessment for the QMG and eyelid drop as reported in the MG-ADL score. From this graph, one observes that only the most extreme scores 0 and 3 are clearly in correspondence. One can also appreciate that the stochastic maps from the MGTX and BeatMG data sets have similarities but are not identical. The variability of the intermediate scores is best described by the probability distribution. Figure 6 represents the stochastic map we obtained for double vision/diplopia. The direct map is close to a bijection, while the inverse map has the tendency to underscore the MG-ADL value. Both stochastic maps are easier representations to interpret than the underlying row distribution of Figure A2. Similar results can be obtained for the other available individual tests that have a cross-correlation value above 0.5 as appreciated earlier—see Figure 4.

**Figure 7:**
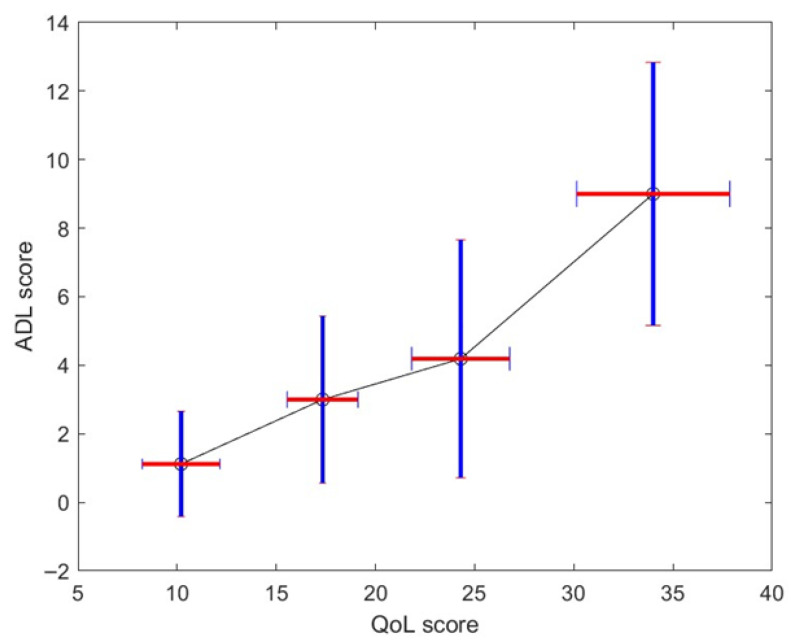
Average and standard deviation—vertical bar—of MG-ADL score for each identified QoL patterns. The horizontal bar represents the mean and average QoL score for each cluster.

**Figure A1:**
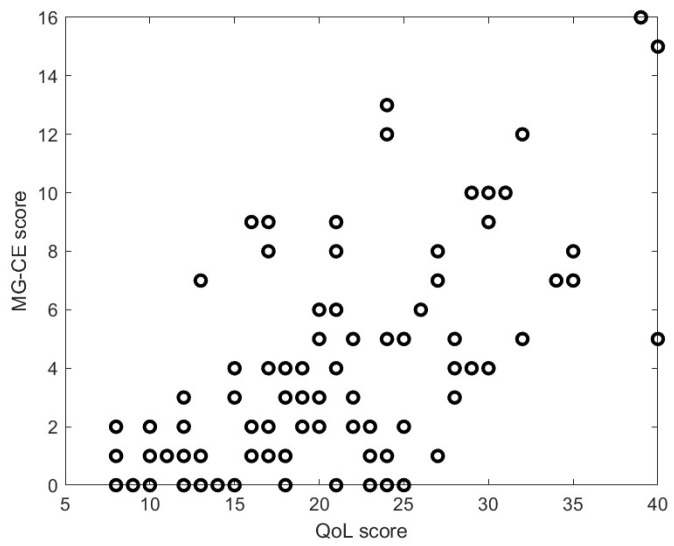
Global MG-ADL score versus QOL score.

**Figure A2:**
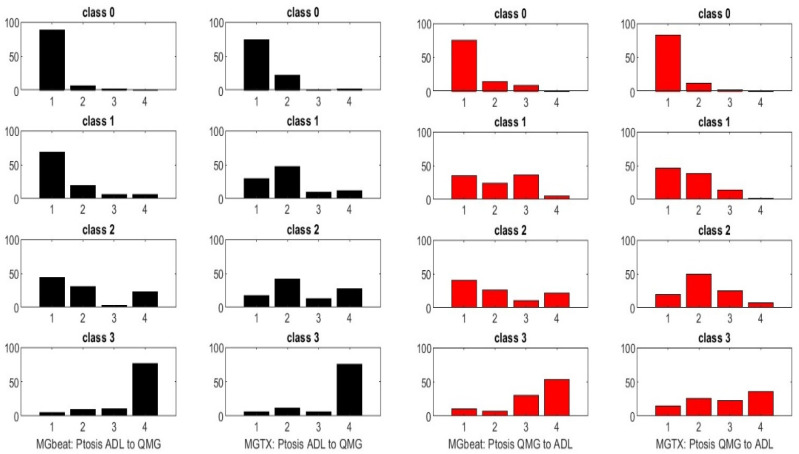
Considering two scores, A and B, one can compute the percentage distribution of score sign in B from a given score in A—see left panel—or vice versa—see right panel. In this graphic, A and B are, respectively, the MG-ADL and QMG scores.

Step 4: Figure 7 shows the stochastic map between QoL and MG-ADL. A similar map was obtained for QoL and QMG. It must be appreciated that the mapping is nonlinear and is monotonic. It is straightforward to obtain a monotonically increasing map: if we use more clusters in the classification of QoL patterns, the map is no longer monotonic because the stage overlaps too much. If we use less, we lower the overlap between the stochastic map values—see vertical bar of Figure 7. While there is a moderate correlation between the MG-ADL score and QoL score with ρ = 0.61 and *p* = 0.4 × 10^−12^, Figure 4 gives us a stochastic map that is more structured than the global score correspondence one—see Figure A1 and take into account the correlation between individual QoL test scores.

Step 5: Overall, we can complete a uniform score portrait of a patient with individual test scores with MG-ADL, QMG, and QoL whenever a stochastic map is available along with its corresponding input is available.

We implemented that strategy on the MGTX patient population and derived for all patients the QoL score from the MG-ADL one, based on the stochastic map statistical elements shown in Figure 7. MG-ADL corresponds to patient report and is therefore appropriate to use for the QoL as opposed to the QMG, which is a single-time-point examiner-generated score. More precisely, assuming a normal distribution, we could generate from the mean and standard deviation on the QoL score corresponding to each level, 1 to 4, of the MG-ADL score.

The score portrait can be optimized further once we filter out from the score all the individual test scores that have the most uncertainty or reliability, like the cheek puff in MG-CE, or use weighted combination that increases the predictability of patient trajectory as defined previously [18].

For now, we use the global (MG-ADL, QMG, and QoL) triplet as the score portrait as a simplification and concentrate on the MGTX data set that had the best success in providing robust results.

To generate a virtual population from that data set of score portraits (MG-ADL, QMG, and QoL) for each patient of the MGTX clinical study, we used a standard Montecarlo method. We assume that the uncertainty on the MG-ADL and QMG score is given by a normal distribution of standard deviation being one, which seems to be a conservative estimate [13]. However, we should further assess the sensitivity of the virtual population that is generated versus that assumption on the standard deviation. Using a scaling factor of 10 with the Montecarlo method, the virtual population has about 1110 patients.

**Figure A3:**
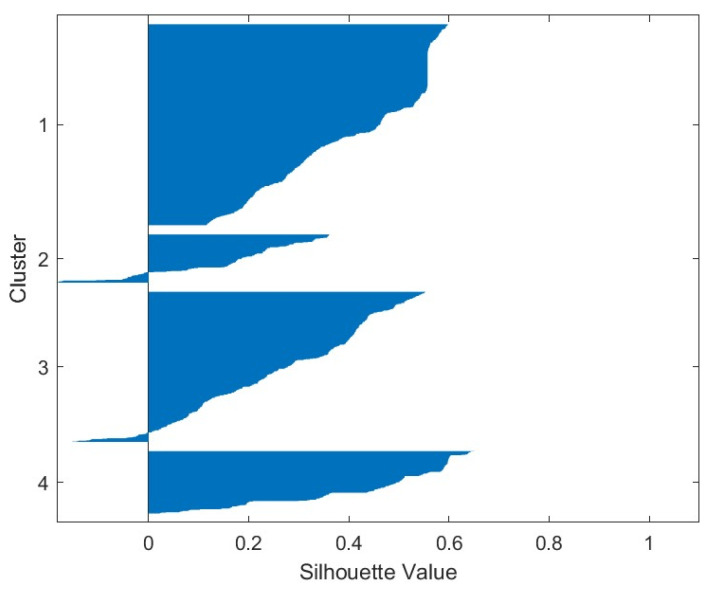
Silhouette map for the clustering of the score portrait of the virtual population of 1110 individuals.

Following the clustering technique described in [16], we compute the mean trajectories of these virtual patients with four clusters and keep a good separation between clusters as shown in the silhouette portrait as shown in Figure A3. This new result seems to bring additional fine details that were not available with the original MGTX data set, where we found that three clusters was the largest number allowed according to the signature function.

**Figure 8:**
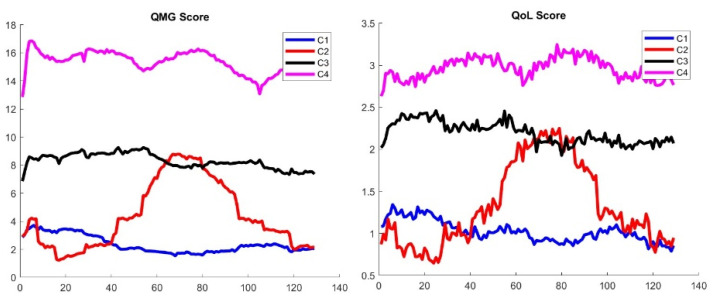
Classification of trajectories for the QMG (**left panel**) and QoL score (**right panel**) of the virtual population generated from the MGTX patients. The QoL score has been generated from the MG-ADL score using the stochastic map of Figure 7.

**Figure A4:**
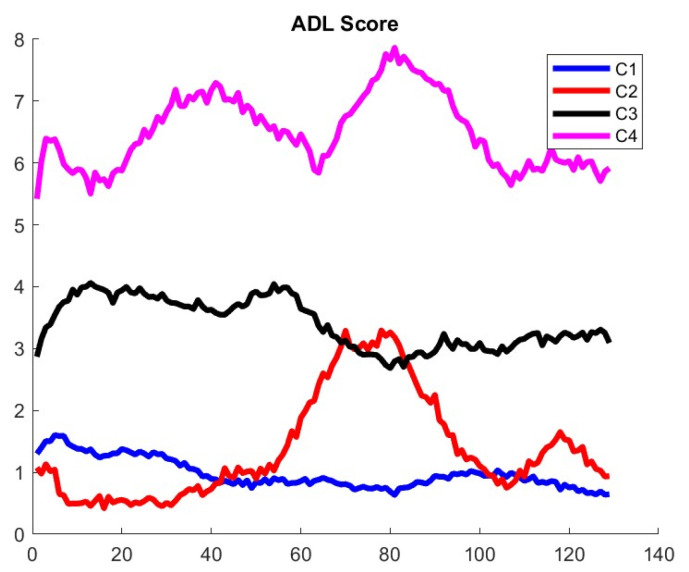
Classification of trajectories for the MG-ADL score of the virtual population generated from the MGTX patient. Figure 8 and Figure A3 provide all together the completed score portrait of these patients.

We can generate a virtual population with QoL pattern score for the MGTX patients that do not have QoL records. Figure 8 shows the main trajectories for the QMG and QoL scores. For completeness, the MG-ADL score trajectories are shown in Figure A4 of Appendix A.

**Figure A5:**
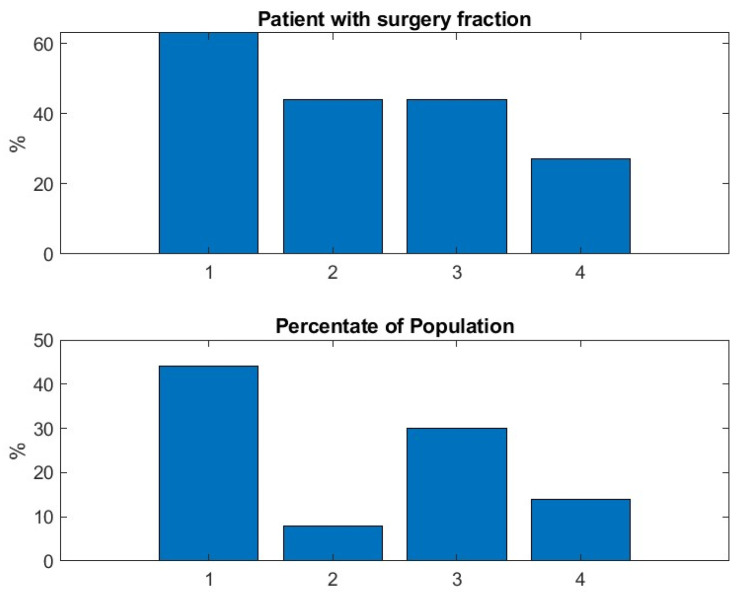
Result confirms the benefit of thymectomy for patient outcome expressed as daily prednisone dose. The thymectomy patients were more common in cluster 1, which identified the subjects who responded best to treatment.

**Figure A6:**
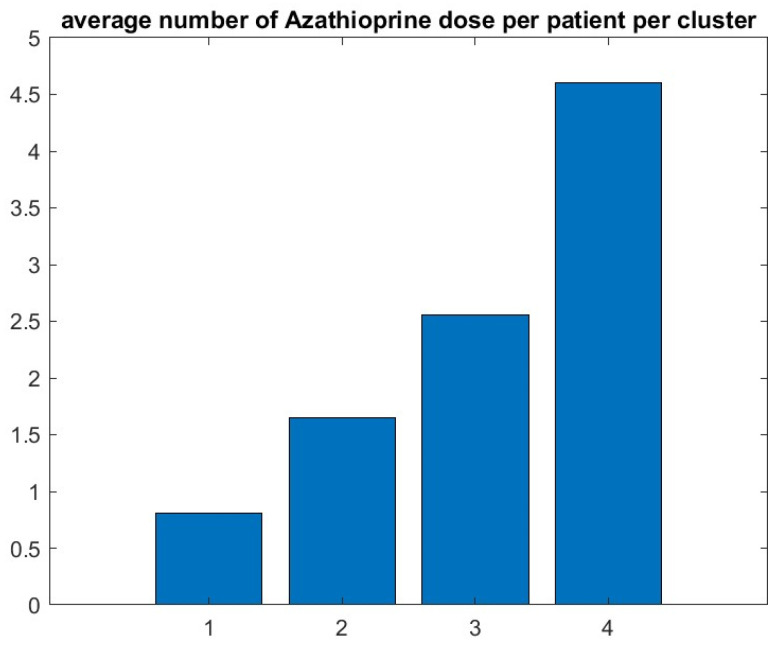
Graphic confirms that the patients who do best (respectively, worse) have the lowest (respectively, highest) number of doses of azathioprine.

It should be appreciated that the clustering was applied to the score portrait, including all three scores at once, instead of each individual score. It is then remarkable that the ranking of clusters from greatest improvement—cluster 1—to treatment resistant—cluster 4—matches the same order for all three scores. While the virtual population offers a much larger sample to cluster, it is not clear why clustering should work better than the original MGTX set. However, the following results provide some validation of the results:

Figure A5 in Appendix A shows the benefit of thymectomy for patient outcome expressed as daily prednisone dose, which was demonstrated by the clinical trial. As expected, thymectomy patients were more common in cluster 1, which identified the subjects who responded best to treatment. Figure A6 graphics confirm that the patients who do best have the lowest number of doses of azathioprine, and conversely, the treatment-resistant patients had the highest use of azathioprine.

**Figure 9:**
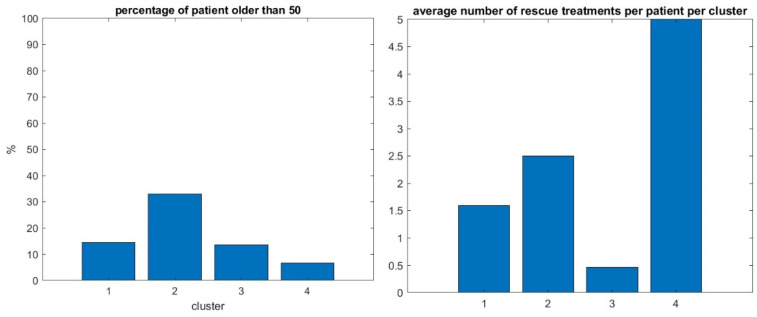
Percentage of patients over 50 in the same four clusters (**left panel**), number of rescue therapy sessions per patient per cluster (**right panel**).

In addition, we observe a new phenomenon with cluster 2 that represents only 7% of the total virtual population. For those patients, the mean trajectory differs significantly from the three others that stay relatively flat. We observe that cluster 2 has a significantly higher percentage of patients older than 50—see Figure 9, left panel.

In our previous study with the MGTX data set alone, i.e., not the virtual population using the score portrait generated here, those individuals over 50 were included in cluster one, which was the largest. We hypothesize that the benefit of generating a virtual population with a score portrait that includes all three scores provides finer granularity that can identify this kind of singular behavior. It remains to be seen if the daily drug dosage could have been managed differently for those patients. The successful isolation of the unique cluster 2 (older, treatment-resistant patients) is our most interesting result; the distribution of patients over the four clusters of this virtual population who went through rescue therapy, i.e., plasmapheresis and intravenous immunoglobulin, confirms that cluster four, that corresponds to patient with poorest outcomes with the less successful therapy, has indeed the greatest use of rescue therapy—see Figure 9, right panel. But since the proportion of patients having rescue therapy is relatively small, i.e., 14%, it will take a larger cohort to better understand the factors producing these clusters.

## 4. Discussion

We have successfully developed a step-by-step method to create a score portrait for patients with MG. This portrait integrates all available information from the QMG, MG-ADL, and MG-QoL scores, drawing from a representative set of independent clinical trial datasets. A key challenge was managing studies with incomplete records or missing scores. The trade-off for this comprehensive construction is that the score portrait is no longer a single number, but rather a probability distribution. The primary advantage of this approach is its ability to generate a uniform metric across various clinical studies, even those not initially designed with matching protocols. However, this comes with a level of uncertainty embedded in the score portrait, which reflects the inherent limitations when information is missing. This concept is broadly applicable to diverse clinical studies beyond just MG. While this feasibility study employed relatively simple statistical approaches, such as stochastic maps between score types or subsets of score types, it clearly demonstrated that none of the individual scores are ideal; for instance, correlations between QoL test values can be strong. To some extent, correlations with muscle groups in QMG, MG-ADL, or MG-CE are non-negligible. To define a more robust score that effectively combines physical examination and patient outcomes—as our MG-ADL, QMG, and QoL combination aims to do—further clinical information data and validation are essential to accurately predict patient trajectories [23,24].

Generating virtual populations of patients from numerous independent clinical trials is a promising strategy to address the challenge of MG’s rare disease status. Clinical trials are compromised by recruitment challenges which slow completion and add cost. In addition, the field has seen significant growth in the number of trials, making for a competitive market for subjects. The option to build a synthetic placebo control arm provides great opportunity for enhancing trial performance. The practical value of these virtual populations has yet to be demonstrated robustly [20]. However, our preliminary results, built on the rigorously performed and complex MGTX study, suggest that exploring virtual populations can reveal finer details on patient trajectories within small subgroups. These insights might otherwise be missed in standard analyses applied to clinical trials. We intentionally kept our algorithmic approach, from step 1 to 5, systematic. This provides an opportunity to re-evaluate current standards for scores and minimum manifestations. Nevertheless, gaps likely exist in clinical study protocols, particularly concerning the impact of patient personality traits that impact patient-reported outcome measures, for example, openness, resilience, and adaptability to disability. These are a crucial component of how patients cope with the disease and interact with clinicians during evaluations.

In addition to clinical trials, the development of comprehensive computational models of patients in the clinical setting would make for a profoundly powerful tool for clinicians. At present, there are no biomarkers that aid in therapy selection for a clinician. At the time of initial visits, the physician has no ability to predict how the individual patient will respond to therapy. As appreciated here and in our other work, we were able to identify a cluster of subjects with a poor clinical score portrait. A clinician with this a priori knowledge would be motivated to monitor these patients more frequently and escalate treatment options earlier.

## 5. Conclusions

This study successfully developed a novel “Myasthenia Gravis Portrait” that integrates various disease severity scores (QMG, MG-ADL, QoL) into a unified, probability-based metric. This approach addresses the challenge of diverse scoring systems and incomplete data across different clinical trials, enabling a more standardized assessment of disease progression. The creation of this portrait facilitates the generation of virtual patient populations, a crucial step for applying machine learning techniques to better understand and predict individual patient trajectories in this rare disease. As mentioned earlier, we need to integrate three primary data types from multiple clinical studies: (i) laboratory and medication data, (ii) Electronic Health Record (EHR) data (e.g., age, sex, years since diagnosis, BMI), and (iii) disease severity scores. The present clinical score portrait is only part of the “patient state” in a clinical study and should eventually include many other factors. The incorporation of additional patient assessment factors, like psychological state and objective fatigue measures, seems a viable future direction of research. While further clinical validation is needed, this framework offers a promising path towards advancing clinical trial performance and ultimately clinical care.

## Figures and Tables

**Table 1:** Hypothetical example of score values using a combination of measured outcome for QMG and estimated outcome for MG-ADL.

**Score Value**	0	1	2	3
**QMG**	0	1	0	0
**MG-ADL**	0.6	0.3	0.1	0

**Table 2:** Hypothetical example of the output of the stochastic map provides a probability distribution for the couple of possible score evaluations (Score A, Score B).

**Test Score A/Test Score B**	0	1	2	3
0	0.1	0.1	0	0
1	0.5	0.2	0	0
2	0	0	0.1	0
3	0	0	0	0

**Table 3:** List of individual test scores for each outcome measure.

Score	List of Individual Test Score
MG-ADL	Talking/chewing/swallowing/breathing/brush teeth/sit to stand/double vision/eyelid drop
MG-CE	Ptosis/diplopia/cheek puff/tounge to cheek/count to 50/arm fatigue/single breath/sit to stand
QMG	Left leg/right leg/head/left grip/right grip/vital/left arm/right arm/speech/swallow/facial/ptosis/vision

**Table 4:** Organization of symptoms from scores by group.

Group 1	Group 2	Group 3	Group 4	Group 5	Group 6
Ptosis	Arm Strength	Breath	Sit to Stand	Talking	Chewing
Diplopia	Arm Extension	Single Breath Count	Leg Strength	Count to 50	Swallowing
Double Vision	Brush Teeth				Tongue to Cheek
Eyelid drop	Grip				Talking
					Facial

## Data Availability

Data supporting the findings of this study are derived from the following sources: ADAPT-teleMG (NCT05917184): Data available upon reasonable request from the study investigators at The George Washington University. MGTX and BeatMG data was made available by request to the National Institute of Neurological Disorders and Stroke. No new human data were generated specifically for this analysis. Restrictions apply to the availability of individual-level data due to patient privacy considerations.
